# Editorial: New methodological, intervention and neuroscientific perspectives in sports psychology, volume II

**DOI:** 10.3389/fpsyg.2025.1588276

**Published:** 2025-04-08

**Authors:** Antonio Hernández-Mendo, M. Teresa Anguera, Verónica Morales-Sánchez, José María Caramés Tejedor

**Affiliations:** ^1^Faculty of Psychology, University of Málaga, Málaga, Spain; ^2^Faculty of Psychology, Institute of Neurosciences, University of Barcelona, Barcelona, Spain

**Keywords:** mixed methods, intervention, neurosciences, sport psychology, neuroscience

This Research Topic presents contributions that expand the frontiers of Sport Psychology, addressing new methodological, intervention, and neuroscientific perspectives. The articles included encompass a broad range of approaches and emerging perspectives, spanning from conceptual research to innovative empirical studies.

At a conceptual level, most articles focus on the relationship between personality variables and sports practice. Several studies delve into the examination of anxiety and insomnia in athletes and their impact on performance. From a methodological standpoint, various investigations employ Mixed Methods, integrating questionnaires with observational techniques, along with neuroscientific tools such as electroencephalography (EEG) and eye-tracking, providing deeper insights into cognitive and emotional processes in sports.

Neuroscience plays a crucial role in this volume, with articles examining the neurophysiological correlates, with fMRI, to investigate brain activity in sport practices such as Baduanjin. A neuropsychological approach is also employed to predict pre-competition anxiety and enhance intervention strategies in high-performance settings.

Through the application of advanced statistical tools and software such as SPSS, JASP, and EEGlab, these studies contribute significant methodological richness, offering valuable resources for future research and interventions in sport psychology. Collectively, these works provide a comprehensive and up-to-date perspective on the latest trends in the field, with the potential to enrich our understanding of mental and emotional processes in sports.

This volume reflects the dynamic evolution of the discipline, and we hope it serves as a valuable resource for both researchers and practitioners interested in sport psychology and its practical applications.

To provide a comprehensive overview of the contributions included in this volume, we present a summary based on different criteria:

## 1 Conceptual scope

Majority of the articles (11) are original research, one focuses on a protocol (Luo et al.), and one is strictly conceptual in nature (Tossici et al.). This Research Topic includes reference works exploring personality variables and their relationship with sports practice (Pineda-Espejel et al.), including tactical decision-making in basketball (Díaz-Rodríguez and Pérez-Córdoba) and gym-goers' behavior (Tavares et al.). Some studies analyze personal motivation and emotion, examining their relationship with academic performance and sports practice (Fierro-Suero et al.). Other focus on methodological aspects, comparing self-perception and observational methodologies in handball (Prudente et al.). Several contribute to scale validation studies in Chinese athletes, specifically on anxiety (Zhang et al.) and non-clinical insomnia (Tan et al.), or in Spanish-speaking Mexican athletes through the validation of the Interpersonal Behavior Questionnaire (IBQ) (Pineda-Espejel et al.). Finally, several articles incorporate a neuroscientific perspective, examining the relationship between sports skills and cognitive/emotional functions through direct neural activity recordings (Carey et al.; Wang et al.; Yu et al.). Additionally, one study proposes a protocol for neuroimaging research (Luo et al.), while another focuses on neuropsychological aspects of sports performance (Caramés et al.).

## 2 Methodological

The empirical studies included in this Research Topic demonstrate different procedural orientations. The majority use questionnaires, either exclusively (Fierro-Suero et al.; Pineda-Espejel et al.; Tavares et al.; Tan et al.; Zhang et al.) or in combination with other methodologies (Caramés et al.; Díaz-Rodríguez and Pérez-Córdoba; Prudente et al.; Yu et al.), including mixed-methods framework (Prudente et al.).

Additionally, one study employs structural equation modeling (Fierro-Suero et al.), while another incorporates standardized tests (Díaz-Rodríguez and Pérez-Córdoba). Three studies primarily focus on adapting questionnaires for different athlete populations, including Chinese athletes (anxiety: Zhang et al.; non-clinical insomnia: Tan et al.) and Mexican athletes (Interpersonal Behavior Questionnaire – IBQ: Pineda-Espejel et al.).

Some studies incorporate cognitive or practical tasks (Caramés et al.; Carey et al.; Wang et al.; Yu et al.) in combination with neuroscientific techniques, such as electroencephalography (EEG) (Carey et al.; Wang et al.; Yu et al.) and eye-tracking (Carey et al.).

This Research Topic includes various software tools and analytical methods relevant to sport psychology.

Regarding software, the studies utilize SPSS, JASP (Carey et al.), and R (Zhang et al.) for statistical analysis. Eye-tracking tools such as ASL X6 Mobile Eye Tracker and EyeVision (Carey et al.) are also employed, along with observational platforms like Lince, SDIS-GSEQ, and Hoisan (Prudente et al.). Neuroscientific research incorporates NeuroScan NuAmps, EEGlab for MATLAB (Yu et al.), ANT Neuro, BioTracer+, and Nexus-10 (Wang et al.). Other specialized platforms include MENPAS (Caramés et al.), REDCap (Tavares et al.), and Mpus (Fierro-Suero et al.; Pineda-Espejel et al.).

The statistical and analytical methods used encompass *t*-tests (Díaz-Rodríguez and Pérez-Córdoba; Fierro-Suero et al.; Yu et al.), ANOVAs (Carey et al.; Yu et al.; Wang et al.), and MANOVAs (Wang et al.). Regression models (Díaz-Rodríguez and Pérez-Córdoba; Fierro-Suero et al.; Zhang et al.), correlation matrices (Díaz-Rodríguez and Pérez-Córdoba; Caramés et al.), and Spearman correlations (Caramés et al.; Prudente et al.) are also applied. Psychometric evaluations include Cronbach's alpha (Caramés et al.; Tan et al.), McDonald's omega (Prudente et al.; Pineda-Espejel et al.; Tan et al.), and confirmatory factor analysis (Pineda-Espejel et al.). Neuroscientific analyses focus on EEG signal processing (Carey et al.; Yu et al.; Wang et al.), power spectrum analysis (Wang et al.), and independent component analysis (Carey et al.; Yu et al.). Additionally, some studies implement machine learning techniques, such as decision trees using CHAID (Tavares et al.).

This Research Topic presents a diverse set of methodologies, statistical techniques, and neuroscientific tools applicable to sport psychology, providing valuable insights for future research and interventions in the field.

## 3 Scope of application

Articles published in this Research Topic present a particular focus on personality factors influencing physical activity (Díaz-Rodríguez and Pérez-Córdoba; Fierro-Suero et al.; Pineda-Espejel et al.; Tavares et al.), with some studies applying these concepts to specific sports, such as basketball (Díaz-Rodríguez and Pérez-Córdoba). Others examine neurophysiological correlates of specific sport tasks, including golf (Carey et al.; Yu et al.), or sport-related skills such as visuomotor ability (Wang et al.).

In addition, a mixed-methods approach is used to increase data reliability, applied to specific situations in handball (Prudente et al.). The neuropsychological approach is also employed to predict anxiety in athletes prior to sporting events (Caramés et al.). Furthermore, three articles focus on the adaptation or validation of questionnaires for use with Chinese athletes (anxiety questionnaire, Zhang et al.; non-clinical insomnia, Tan et al.) or Mexican athletes (IBQ, Pineda-Espejel et al.).

This Research Topic also includes a conceptual work that focuses on the Psiconeuroendocrinological (PNEI) approach to stress phenomena in sports performance, providing a holistic perspective (Tossici et al.). Moreover, a protocol is presented for implementing fMRI in sport psychology, specifically in the practice of Baduanjin (Luo et al.).

In summary, we highlight the contributions of the 13 articles published in this Research Topic and offer them to our readers, with the hope that they will help expand knowledge in this field.

## 4 Conclusions

The articles included in this Research Topic provide a comprehensive range of theoretical and methodological contributions. As editors, we are pleased to present these works and their valuable transfer to the scientific community.

The articles in this volume have garnered significant attention, as evidenced by their high number of views, according to internal metrics. All of the articles were published between 2023 and 2024. Of the 26 articles initially submitted, 13 were ultimately accepted, yielding an acceptance rate of 50%.

This Research Topic reflects the evolving landscape of Sport Psychology, and we are confident that it will make a substantial impact in advancing knowledge and informing future research in the field.

